# Evaluation of Differentiated Bone Cells Proliferation by Blue Shark Skin Collagen via Biochemical for Bone Tissue Engineering

**DOI:** 10.3390/md16100350

**Published:** 2018-09-25

**Authors:** Jeevithan Elango, Jung Woo Lee, Shujun Wang, Yves Henrotin, José Eduardo Maté Sánchez de Val, Joe M. Regenstein, Sun Young Lim, Bin Bao, Wenhui Wu

**Affiliations:** 1 Department of Marine Bio-Pharmacology, College of Food Science and Technology, Shanghai Ocean University, Shanghai 201306, China; srijeevithan@gmail.com (J.E.); truthwo@naver.com (J.W.L.); 2 Division of Marine Bioscience, Korea Maritime and Ocean University, Busan 606791, Korea; sylim@kmou.ac.kr; 3 Co-Innovation Center of Jiangsu Marine Bio-industry Technology, Huaihai Institute of Technology, Lianyungang 222005, China; shujunwang86@163.com; 4 Bone and Cartilage Research Unit, Arthropôle Liège, University of Liège, CHU Sart-Tilman, 4000 Liège, Belgium; yhenrotin@ulg.ac.be; 5 Department of Biomaterials Engineering, Universidad Católica San Antonio de Murcia, 30107 Murcia, Spain; jemate@ucam.edu; 6 Department of Food Science, Cornell University, Ithaca, NY 14853-7201, USA; jmr9@cornell.edu; 7 Laboratory of Quality and Safety Risk Assessment for Aquatic Products on Storage and Preservation, Ministry of Agriculture, Shanghai 201306, China

**Keywords:** blue shark collagen, osteogenic activity, Runx2, differentiated mesenchymal stem cell, osteoblast, proliferation

## Abstract

Collagen from a marine resource is believed to have more potential activity in bone tissue engineering and their bioactivity depends on biochemical and structural properties. Considering the above concept, pepsin soluble collagen (PSC) and acid soluble collagen (ASC) from blue shark (*Prionace glauca*) skin were extracted and its biochemical and osteogenic properties were investigated. The hydroxyproline content was higher in PSC than ASC and the purified collagens contained three distinct bands α_1_, α_2,_ and β dimer. The purity of collagen was confirmed by the RP-HPLC profile and the thermogravimetric data showed a two-step thermal degradation pattern. ASC had a sharp decline in viscosity at 20–30 °C. Scanning electron microscope (SEM) images revealed the fibrillar network structure of collagens. Proliferation rates of the differentiated mouse bone marrow-mesenchymal stem (dMBMS) and differentiated osteoblastic (dMC3T3E1) cells were increased in collagen treated groups rather than the controls and the effect was dose-dependent, which was further supported by higher osteogenic protein and mRNA expression in collagen treated bone cells. Among two collagens, PSC had significantly increased dMBMS cell proliferation and this was materialized through increasing RUNX2 and collagen-I expression in bone cells. Accordingly, the collagens from blue shark skin with excellent biochemical and osteogenic properties could be a suitable biomaterial for therapeutic application.

## 1. Introduction

There are three major types of protein present in fish, such as sarcoplasmic (25–30%), myofibrillar (66–77%), and stroma protein (1–5%). Among them, stromal proteins are mostly located in the interstitial space of muscle cells and the extracellular membrane. One of the most available stromal proteins in fish is collagen. It is extracted from different part of fish such as bones, scales, skin, swim bladder, and fins. Currently, collagen from the marine organism has been widely investigated due to its excellent biological activity with low or no side effect as an alternative to mammalian collagen [1,2]. Collagen is graded based on its biochemical, functional, and rheological characteristics, which may depend on fish species and the manufacturing method. Based on the quality and bioactivity, fish collagen can be used as a drug in the biomedical industries or as a food supplement in food industries.

In tissue engineering, fish collagen is used in the form of films, scaffolds, sponge, hydrogel, microspheres, and composite biopolymers. Wang et al. [3] studied the hemostatic properties of type I collagen to treat tissue burns. In another study, type I collagen 3D matrix was used as a potential biomaterial for heart regeneration [4]. It is well known that the growth and characteristic of cells such as proliferation, adhesion, and maturation were enhanced by collagen labeling [5]. It was found that the inflammatory response of tilapia collagen biomaterials in rabbits was similar to that of collagen from porcine or polyethylene [6]. In addition, the collagen from jellyfish could induce an immune response similar to that of bovine collagen [7]. Sugahara et al. [8] reported that jellyfish collagen produced by pepsin digestion up-regulated the production of immunoglobulin IgM in the human hybridoma cells and IgM and IgG in human peripheral blood lymphocytes due to the presence of telopeptides. Our recent research confirmed the biocompatible and non-immunogenic effect of acid soluble- and pepsin soluble-collagen isolated from shark cartilages and tilapia skin and these collagens did not elicit immune response in in-vivo and in-vitro models [9,10]. In that sense, collagen from shark can be used as a potential biomaterial for bone tissue engineering.

Indeed, shark collagen has been widely used in bone tissue engineering application due to its excellent biocompatible, osteoconductive, osteoinductive, and natural bone biomimetic properties. The collagen material used in bone tissue engineering not only regulates morphological properties but also maintains appropriate cues for regulating cellular processes during bone formation. It was reported that the collagen-based biomaterial implanted in rabbit induced neotendon and neoligament formation [11,12]. Our previous study revealed the osteogenic potential of whale shark bone collagen on mesenchymal stem cells and primary osteocytes [13]. In another study, composite scaffold 3D matrices were prepared by mixing acid soluble collagen from blue shark (*Prionace glauca*) skin with calcium phosphates from the teeth of *Prionace glauca* and *Isurus oxyrinchus* and tested the effect of a composite collagen scaffold 3D matrix in the proliferation of osteoblast-like cells, Saos-2 [14]. However, the osteogenic properties of pure collagen isolated from blue shark skin need to be addressed before being used as a biomaterial in bone tissue engineering, since the osteogenic potential of fish collagen is profoundly influenced by the molecular composition and arrangement, which is thought to be varied by different extraction methods. Considering the above hypothesis, for the first time, we explored the osteogenic response of type I collagens (Acid and Pepsin soluble) isolated from blue shark (*Prionace glauca*) skin using differentiated mouse bone marrow-mesenchymal stem (dMBMS) and differentiated osteoblastic (dMC3T3E1) cells and their relationship with the biochemical and functional properties of collagen in the present study.

## 2. Results and Discussion

### 2.1. Hydroxyproline and Collagen Content of Raw Materials

**Figure 1:**
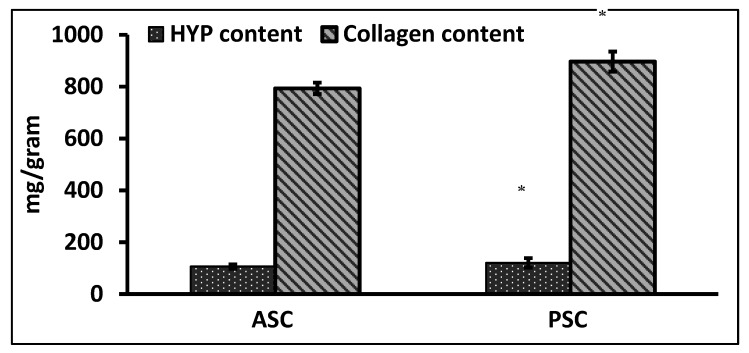
Hydroxyproline and collagen content of blue shark skin collagen. ASC-Acid soluble collagen, PSC-Pepsin soluble collagen. * *p* < 0.05 vs. ASC.

The quantification of total collagen content from hydroxyproline (Hyp) was determined as per the earlier method [15]. In general, the PSC had a high content of Hyp than ASC, similarly the collagen content was also high in PSC. The hydroxyproline and collagen contents of PSC and ASC were about 120 and 106.36 mg/g and, 896.738 and 793.49 mg/g, respectively (Figure 1). The present result was in accordance with the hydroxyproline content of collagen from silvertip shark (113 and 105 mg/g) and brownbanded bamboo shark (103.71 mg/g) [1,16]. The hydroxyproline content may depend on the seasonal variations and body temperature of the fish species.

### 2.2. Amino Acid Content

The amino acid profiles of blue shark collagens (PSC and ASC) were similar in trend and expressed as residues per 1000 total residues (Table 1). Both ASC and PSC had glycine (392 and 387) as a foremost amino acid, followed by alanine (144–130) and proline (132–124). The amino acid contents of glycine, asparagine, proline, and hydroxyl-proline were high in general. The sum of Pro and Hyp (imino acid) content was higher in PSC (222) than ASC (213). The high content of alanine, glycine, hydroxyproline, and proline were the typical amino acids that represent the high content of collagen in blue shark skin. The present finding of collagen amino acid composition was similar to other fish collagen [16,17]. The differing amino acid composition between PSC and ASC was due to the removal of some non-collagen amino acid and the breakdown of certain specific amino acid residues at the telopeptide region during pepsin hydrolysis.

### 2.3. Molecular Weight Analysis

**Figure 2:**
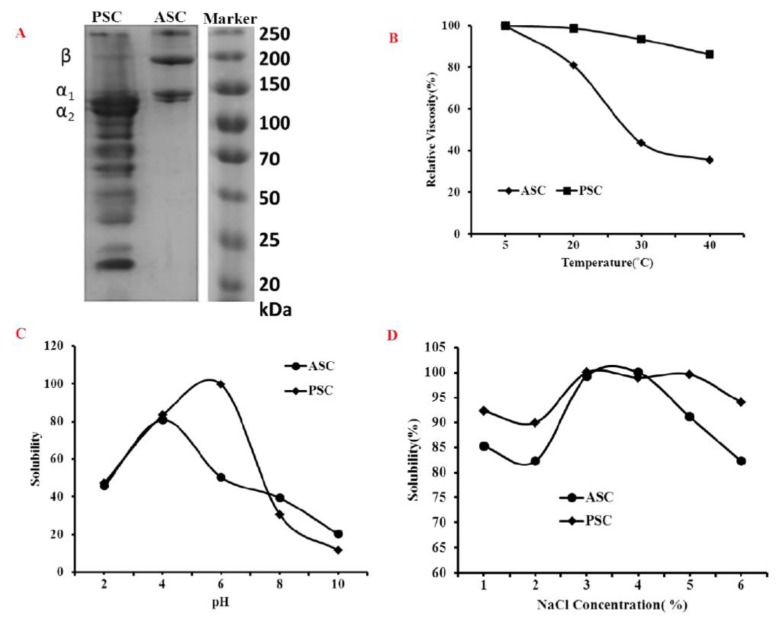
Electrophoretic profile (**A**), viscosity (**B**) and solubility against pH (**C**) and salt (**D**) of blue shark skin collagen. ASC-Acid soluble collagen, PSC-pepsin soluble collagen.

The electrophoretic pattern of the skin collagen was shown in Figure 2A. The electrophoretic pattern of ASC and PSC was comparable, which was comprised of three distinct bands, α_1_, α_2_ and β chains. However, there was some smaller molecular weight component observed in the PSC electrophoretic pattern, which was due to the hydrolysis of pepsin at the telopeptide region. The present finding of the collagen molecular pattern (α_1_, α_2_ and β) was a characteristic type I collagen electrophoretic profile and was comparable with other teleost fish species [18].

### 2.4. Viscosity and Solubility

Upon increasing temperature, the viscosity of ASC and PSC had decreased gradually (Figure 2B). The viscosity of PSC was gradually decreased in respect to temperature, which was similar to other fish collagen [19,20], whereas the viscosity of ASC had a sharp decline at 20–30 °C. The collagens had lost their viscosity against the temperature due to the breaking of the collagen molecule hydrogen bonds that lead separate triple helix chains into random coils or distinct chains during heating at high temperature. Therefore, the secondary structure highly influences the viscosity of collagen. In this study, PSC was more viscous than ASC due to the high content of imino acid, which is believed to be responsible for the stability of collagen [19].

The solubility of collagen against pH was presented in Figure 2C. The results showed that ASC had high solubility in acidic conditions at pH 4.0, whereas the maximum solubility PSC was observed at pH 6.0. The solubility of collagen against salt, NaCl, showed that the maximum solubility of PSC and ASC was observed at 3% and 4% NaCl concentration, respectively (Figure 2D). Similar findings on salt solubility (3–4%) of eel fish skin collagen were earlier reported by Veeruraj et al. [17]. In general, the collagen tends to precipitate at their iso-ionic point (pH 5–8), which leads to decrease the solubility [18]. Also, the solubility of collagen against pH is closely related with the molecular composition and ionic state of the functional groups present in collagen [15].

### 2.5. UV Absorbance

**Figure 3:**
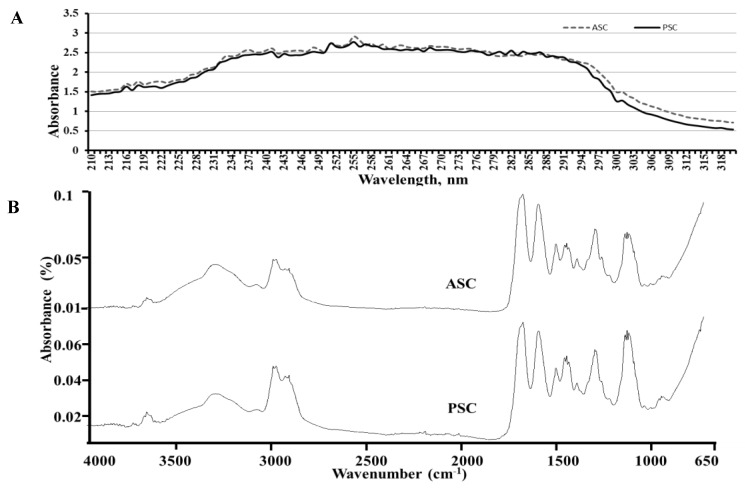
UV absorption (**A**) and Fourier transform infrared spectroscopy (FTIR) pattern (**B**) of blue shark skin collagen. ASC-Acid soluble. collagen, PSC-Pepsin soluble collagen.

The UV absorbance pattern of blue shark skin collagen was presented in Figure 3A. Both collagens, PSC and ASC, had a UV maximum absorption at 225 nm. It was reported that the major functional groups (-COOH, C=O and CO-NH_2_) of collagen tend to absorb UV light [21]. An earlier study revealed that the purity of collagen was indirectly measured by the absence of UV absorption at 280 nm since the non-collagen proteins tend to absorb maxima at this particular UV wavelength [16].

### 2.6. Fourier Transform Infrared Spectroscopy (FTIR)

The FTIR peak of blue shark skin collagen was presented in Figure 3B. As shown, the primary peaks such as Amide-A and Amide-B were observed at 3302.67 and 3301.20 cm^−1^, and 2972.57 and 2972.36 cm^−1^ for ASC and PSC, respectively. Amide-A and Amide-B peaks are generally produced by N-H stretch joined with the H_2_ bond in a carbonyl group of peptide chains and an asymmetric stretching vibration of NH_3_^+^ with =C-H, respectively. The major spectral wavelength observed in blue shark skin collagen was similar to earlier studies [17,20,22,23]. In this study, the shifting of Amide-A peak in PSC to lower wavelength was due to the hydrogen bond vibration of the NH group.

The other major peaks like Amide-1 and Amide-2 occurred at 1632.28–1632.06 cm^−1^ and 1547.89–1547.25 cm^−1^ for blue shark skin ASC and PSC, respectively. The secondary structure of collagen is closely represented by Amide-1 peak, which is due to the C=O stretching vibration of peptides. Therefore, any shifting in the amide-1 peak directly reflects the changes in the secondary structure of a protein. In this study, the amide-1 peak of PSC shifted to a lower wavelength compared to ASC, which denotes the secondary structural changes of PSC by pepsin digestion.

### 2.7. Reversed-Phase High-Performance Liquid Chromatography (RP-HPLC)

**Figure 4:**
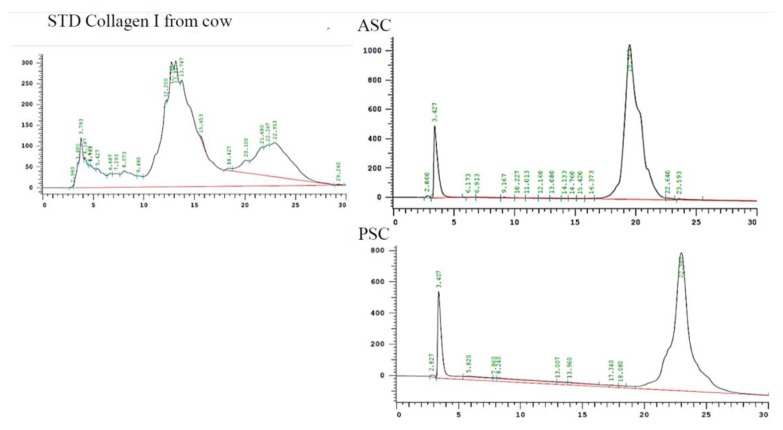
RP-HPLC elution profile of blue shark skin collagen. ASC-Acid soluble collagen, PSC-Pepsin soluble collagen.

In order to confirm the purity, the extracted blue shark skin collagens were eluted through RP-HPLC column and compared with standard bovine collagen. As shown in Figure 4, there were two main chromatogram peaks eluted at 3.427 and 19.0 min for ASC and 3.407 and 22.980 min for PSC, respectively. The first peak observed at 3.4 was the acetic acid peak. The present observation was in accordance with a previous report [24]. Compared to the standard bovine collagen, the blue shark fish collagen showed a dissimilar chromatographic pattern. We speculated that this might be due to the difference in their molecular arrangements between mammalian and fish collagen. Also, the RP-HPLC pattern confirmed that extracted collagen was pure and free from non-collagen protein and other contaminants, further justifying the SDS-PAGE data of collagen (Figure 2A).

### 2.8. Thermal Properties

**Figure 5:**
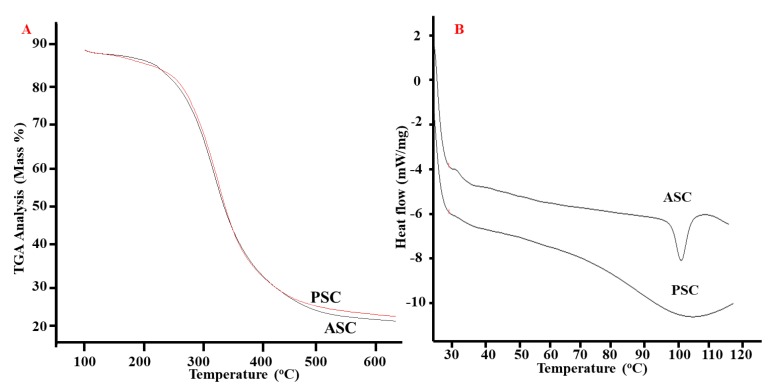
Thermogravimetric analysis (TGA) (**A**) and differential scanning calorimetry (DSC) (**B**) of blue shark skin collagen. ASC-Acid soluble collagen, PSC-pepsin soluble collagen.

Thermal properties such as the material weight loss towards temperature and the thermal disintegration configuration of the blue shark skin collagens were evaluated by the thermogravimetric analyzer (TGA). From the TGA curves, 50% weight loss in ASC and PSC were observed at 339 and 342 °C, respectively (Figure 5A). At high temperature (650 °C), the mass weight of ASC and PSC were about 22.53 and 23.64, respectively.

ASC and PSC from blue shark skin had two endothermic peaks, which denoted the thermal denaturation and melting temperature. The denaturation temperature of ASC and PSC was about 28.3 and 29.8 °C, respectively (Figure 5B). The degree of hydroxylation and certain specific amino acid residues such as Gly-Pro-Hyp might determine the denaturation temperature of collagen [15]. It was observed that imino acids play a major role in the thermal properties of collagen. For instance, the hydroxyproline mainly contributes to the stability of the helix chain [25]. In this study, PSC had better thermal properties than ASC due to the high content of iminoacids.

### 2.9. Morphalogical Characteristics

**Figure 6:**
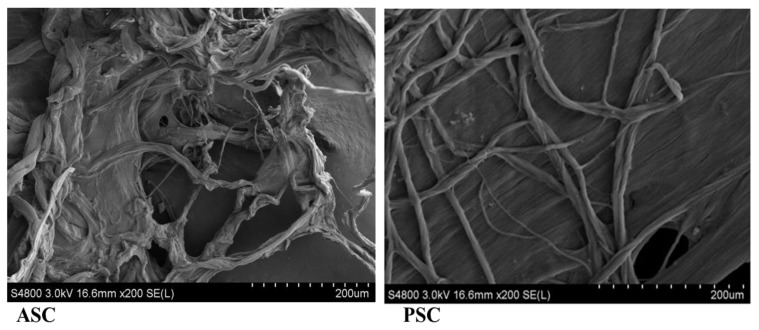
Morphological features of blue shark skin collagens using field emission scanning electron microscope. ASC-Acid soluble collagen, PSC-Pepsin soluble collagen. SEM image with 200 micro meter magnifications.

The field emission scanning electron microscope was used to analyse the morphological structures of the blue shark skin collagens (PSC and ASC). From the microphotography, both collagens were rich in a fibrillar network with many flaky orientation layers (Figure 6). The present finding was in accordance with the morphology of other fish collagens reported earlier [10,17]. However, the morphology was regular and an organized fibrillar network in PSC, while ASC had irregular fibrillar interconnectivity and wall morphology. It was opioned that the morphalogical features of collagen such as fibril interconnectivity, shape and wall morphology were important factors in tissue engineering, since they may influence the growth, migration, proliferation, differentiation, and maturation of cells [26]. In recent research, the collagen from the same species (blue shark skin) was used to formulate a scaffold 3D matrix and tested the proliferation and mineralisation effect on Saos-2 cells, and further confirmed that the scaffold prepared from blue shark skin collagen could support Saos-2 cell attachment and osteoblast-like cells formation [14]. In the present study, the morphological features confirmed the aptness of blue shark skin collagens in bone tissue engineering.

### 2.10. Effect of Collagen on Bone Cells

**Figure 7:**
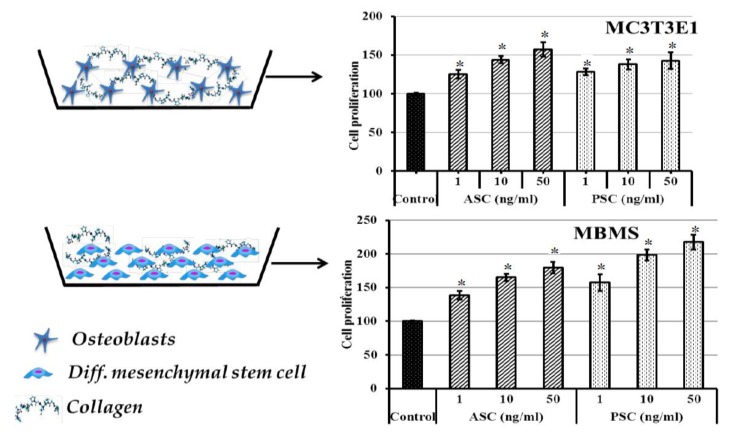
The effect of blue shark skin collagen on differentiated bone cells proliferation. MBMS: differentiated mouse bone marrow mesenchymal stem cells, MC3T3E1- differentiated-osteoblasts, ASC-Acid soluble collagen, PSC-pepsin soluble collagen. For differentiation, MBMS and MC3T3E1 cells were cultured with osteoblast differentiation medium for 21 days. * *p* < 0.05 vs control.

The effect of collagens on the dMBMS and dMC3T3E1 cell growth was determined and presented in Figure 7. As shown in Figure 7, the collagens treated dMBMS and dMC3T3E1 cells showed a higher cell proliferative rate than the control cells and the rate was accelerated with increasing collagen concentration in a dose-dependent manner. Remarkably, the osteogenic effect of the collagen on the bone cell was maxima at a high concentration (50 ng/mL). Among the two cells, dMBMS cells had a higher proliferation rate in PSC treated group. Whereas dMC3T3TE1 cells showed similar cell proliferation in both ASC and PSC treated groups. A recent study by Diogo et al. [14] reported that the composite blue shark skin collagen-calcium phosphate scaffold crosslinked with 12.5% EDC/NHS accelerated Saos-2 cells metabolic activity and supported osteoblast-like cells formation, however, this study was conducted using acid soluble collagen. Conversely, in the present study, the collagen was extracted by two different methods using acetic acid and pepsin, respectively, and the proliferative effect of freeze dried collagens was tested using dMBMS and dMC3T3E1 cells.

**Figure 8:**
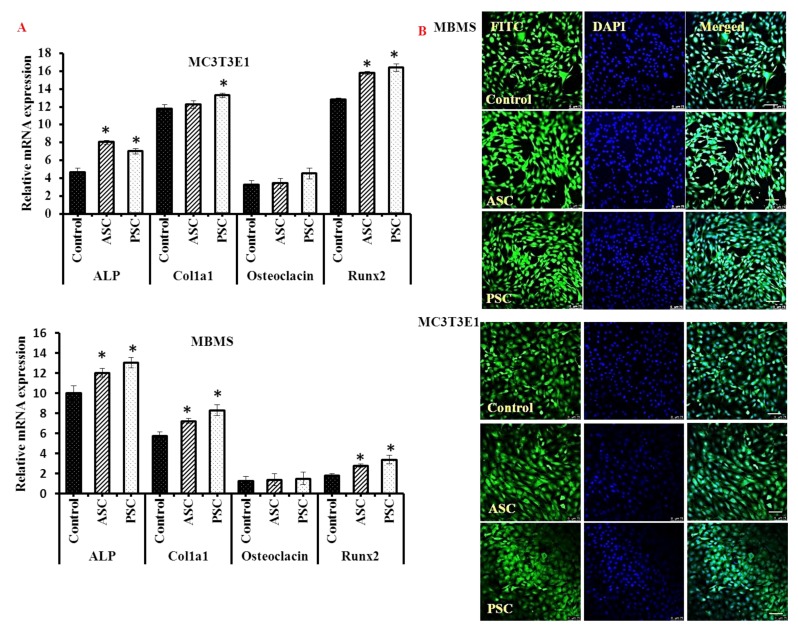
mRNA (**A**) and confocal images (**B**) of blue shark collagen treated bone cells. Scale bars: 75 micrometers. MBMS: differentiated mouse bone marrow mesenchymal stem cells, MC3T3E1- differentiated osteoblasts, ASC-Acid soluble collagen, PSC-pepsin soluble collagen. For differentiation, MBMS and MC3T3E1 cells were cultured with osteoblast differentiation medium for 21 days. * *p* < 0.05 vs. control.

The levels of osteogenic mRNA expression of alkaline phosphatase, collagen 1 aplha1 and Runx2 were significantly increased in the collagen treated cells than the control cells, however, the osteocalcin mRNA expression was not significantly altered between the collagen and control cells (Figure 8A). Confocal laser scanning microscope showed a high number of mature bone cells in collagen treated cells compared to control cells (Figure 8B), which further substantiated the osteogenic stimulatory activities of blue shark skin collagens. Similar to the present study, type I collagen and its peptides from rat tail showed osteogenic stimulatory activities on a mesenchymal stem cell [27,28]. Recently, Chiu et al. [29] reported that the MBMS cell expressed a high the level of integrin α_2_β_1_ complex upon collagen treatment. In support of the proliferation result, the levels of osteogenic mRNA of alkaline phosphatase and collagen 1 were increased in collagen treated cells. Runx mRNA expression of collagen treated cells revealed the potential osteogenic activity of collagen. These findings further justify the increased proliferation rate of collagen treated cells. Recent studies claimed that certain amino acids such as glutamine, alanine, asparagine, and glycine of collagen triggered new bone cell formation through the initiation of FAK-JNK signaling pathway via RUNX2 in MBMS cell [29,30].

**Figure 9:**
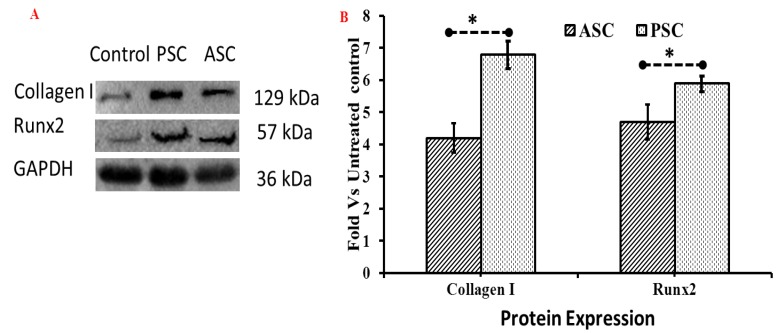
Osteogenic regulatory protein expressions (Collagen I and Runx2) of bone cells (differentiated MBMS) treated with blue shark skin collagen by western blot assay. (**A**) Level of osteogenic protein expression of bone cells (**B**) Fold changes of osteogenic protein expression compared to control (GAPDH). ASC-Acid soluble collagen, PSC-Pepsin soluble collagen. For differentiation, MBMS cells were cultured with osteoblast differentiation medium for 21 days, * *p* < 0.05.

The western blot analysis of the osteogenic protein expression of collagen treated cells was in agreement with the proliferation, mRNA expression, and microscopic results, which indicated the higher osteogenic protein expression of collagen I alpha I and Runx2 in collagen treated cells than in the control (Figure 9A).

In addition, the cells treated with PSC showed higher osteogenic regulatory protein expressions than ASC treated cells (Figure 9B). Interestingly, the Runx2 protein was highly expressed in collagen treated cells, especially in PSC treated cells. It was reported that the collagen could interact with integrin alpha1 beta 2 of the mesenchymal stem cells and trigger FAK/JNK signals through Runx2 during osteoblast differentiation [29]. In that sense, the blue shark skin collagen accrued osteoblast differentiation through upregulating the Runx2 protein expression in dMBMS cells. These findings further confirmed the admirable osteogenic properties of blue shark skin collagens.

## 3. Materials and Methods

### 3.1. Extraction, Purification, and Total Collagen Content of Fish Collagen

The raw material, blue shark skin (*Prionace glauca*), was procured from a private fish processing plant, M/s. Yueqing Biological Health Care Product Co., Ltd. Zhejiang, China and chopped into small pieces. They were then homogenized in PBS prior to extraction. The pretreatment was done in order to remove water-soluble protein, non-collagen protein, and mineral content using distilled water, NaOH and ethylenediaminetetraacetic acid (EDTA), respectively. The pretreated samples were used for the extraction of acid soluble collagen (ASC) and pepsin soluble collagen (PSC) using acetic acid and acetic acid containing 1% pepsin, respectively, as per our previous method [10]. The extracted samples were purified using Sephadex G-100 column chromatography coupled with UV spectroscopy with the absorbance of 230 nm [1]. The purified samples were freeze-dried using a lyophilizer. The hydroxyproline and total collagen content of the purified samples were determined [31].

### 3.2. Molecular Mass by SDS-PAGE and Amino Acid Profile

The protein molecular mass analysis was done by SDS-PAGE using 10% running gel, 4% stacking gel, 20 microliter sample loading volume (10 micrograms per lane) and 120 voltage as per our earlier method [10]. After the run, the gels were stained with Coomassie blue R-250 in methanol and acetic acid for 30 min followed by detaining with methanol and acetic acid [32]. The amino acid profile was determined by hydrolyzing the samples in 6M hydrochloric acid at 110 °C for 24 h and then injected (aliquot of 0.4 mL) into an amino acid analyzer (Hitachi L-8800, Tokyo, Japan) and expressed as residues per 1000 residues [1].

### 3.3. Viscosity and Solubility

The viscosity and solubility of the collagen samples were done as per our earlier method [1]. For viscosity, the samples were heated from 5 to 40 °C followed by injection to a Brookfield LVDV-II+P viscometer (Brookfield Engineering Laboratories Ltd., Middleboro, MA, USA) to measure relative viscosity. The percentage of collagen solubility was tested against different pH (2–10) and salt concentrations (1–6%). The relative solubility was measured from the protein content [33].

### 3.4. Absorbance UV Maxima and FTIR Spectra

The maximum UV absorbance of collagen was done using a UV-spectrophotometer, scanning at a different wavelength from 210 to 318 nm. For the FTIR spectra, the collagen samples were mixed with kBr to produce a disk and scanned at different wavelengths using a Fourier transform infrared spectrometer (Nicolet 6700, Thermofisher Scientific Inc., Waltham, MA, USA) equipped with a DLaTGS detector. The absorption intensity of each peak was calculated as mentioned in our previous method [1].

### 3.5. RP-HPLC

The purity of extracted collagen was confirmed by RP-HPLC as per our earlier method [34]. In brief, a collagen sample dissolved in acetic acid was loaded on a ZORMAX 300 SM-C18 (Agilent, Shanghai, China) column of LaChrom Elite HPLC system (Hitachi, Tokyo, Japan) equipped with an L-2130 pump, L-2300 column oven, L-2400 UV detector and L-2200 auto-sampler; and eluted with a two gradient solvent system using acetonitrile and trifluoroacetic acid with the peak absorbance at 230 nm using the HITACHI D-2000 Elite workstation software (Hitachi, Tokyo, Japan).

### 3.6. Thermal Stability

The thermal stability of collagen was determined from differential scanning calorimetry (DSC) and TGA analysis [35]. In brief, the DSC spectrum was obtained by scanning the collagen samples from 20 to 120 °C using a differential scanning calorimeter (Model-DSC822e, Mettler-Toledo GmbH, Greifensee, Switzerland). For TGA Analysis, the samples were scanned using a TG 209 F1 analyzer (NETZSCH-Geratebau GmbH, Selb, Germany) from 0 to 650 °C at a rate of 10 °C min^−1^ in a nitrogen atmosphere.

### 3.7. Morphological Analysis

The morphology of the blue shark skin colalgens, PSC and ASC was captured using a field emission scanning electron microscope (FE-SEM S-3400 N, Hitachi, Tokyo, Japan) operated at 5 kV. The samples were sputter-coated with gold to produce 10 nm thin layer and moved to the SEM chamber for image capturing at 20 kV voltage.

### 3.8. Effect of Collagen on Osteoblastogenesis

#### 3.8.1. Cell Culture

Mouse bone marrow-mesenchymal stem (MBMS) and MC3T3E1 cells were received from the cell bank (Shanghai Zhong Qiao Xin Zhou Biotechnology Co., Ltd., Shanghai, China) and were cultured in stem cell culture medium and DMEM, respectively, comprising 10% FBS and antibiotics (1% penicillin/streptomycin) at 37 °C in a CO_2_ incubator. Mesenchymal stem cell growth supplement was added with the culture medium to induce MBMS cell growth.

#### 3.8.2. Osteogenic Differentiation

The MBMS and MC3T3E1 cells with a density of 5 × 10^4^ cells/well were seeded in 6 well microtiter culture plate and cultured with osteoblast differentiation medium composed of growth supplements (Shanghai Zhong Qiao Xin Zhou Biotechnology Co., Ltd., Shanghai, China) for 21 days as reported in our previous protocol [13]. The culture medium was replaced every three days.

#### 3.8.3. Proliferation Assay

After differentiation, the cells were trypsinized using EDTA/Trypsin solution and re-seeded in 96 well plates. Then the cells were treated with 1, 10, and 50 ng/mL concentration of blue shark skin collagens. Cells grown without collagen samples served as the control. The proliferative effect of collagen on bone cells was measured using a cell counter (Invitrogen, Countess II Automated Cell Counter, ThermoFisher Scientific, Shanghai, China) at 3 days after treatment.

#### 3.8.4. mRNA Expression

The bone cells were treated with collagen as mentioned above and the total mRNA was isolated using the TRIzol method as per our earlier protocol [13]. Briefly, a Trizol lysed cell monolayer was mixed with chloroform to obtain RNA and the RNA was precipitated using isopropanol. After thorough washing with 70% ethanol, the RNA was converted to first strand cDNA using an Invitrogen kit as per the manufacturer’s instructions. The percentage of osteogenic mRNA expression (ALP, Runx2, osteocalcin, Col1a1, and beta-actin) (Table 2) of blue shark collagen treated bone cells was determined using an ABI 7500 Fast Real-Time PCR System (Applied Biosystems, Shanghai, China) [13]. The cDNA template was mixed with SYBR Green Fast qPCR RT Master Mix (Invitrogen, Shanghai, China) and target primers as per the manufacture’s instructions. The relative mRNA expression of the target gene was calculated by subtracting the mRNA expression of a house-keeping gene, beta-actin.

#### 3.8.5. Immunocytochemistry

For immunocytochemistry, cells were grown in the confocal disc (Cat no. 150682, ThermoFisher Scientific, Shanghai, China) with collagen, washed with ice-cold PBS, fixed with 4% paraformaldehyde for 15 min and permeabilized at room temperature with 0.1% Triton X-100 for 15 min. Then, the cells were incubated with fluorescence stains such as fluorescein isothiocyanate (FITC) and 4′,6-diamidino-2-phenylindole (DAPI). The images were captured using a confocal laser scanning microscope (Leica TCS SP8, Leica Microsystems CMS GmbH, Wetzlar, Germany).

### 3.9. Western Blot Analysis

The osteogenic effect of the ASC and PSC was further tested in dMBMS cells using the western blot method, as an empirical study. In brief, the cells were seeded at a cell density of 1 × 10^5^ in 6 well plates and cultured with 1 mL culture medium for control. For collagen treatment, the samples (50 ng/mL) were mixed with 1 mL cultured medium and cultured for three days. Then, the treated and untreated cells were harvested using a lysis buffer with 30 s sonication and centrifuged at 12,000 rpm at 4 °C for 15 min for the extraction of total cellular protein. The protein content was quantified using a BCA kit as per the manufacturer’s instructions (Thermo Fisher Scientific, Shanghai, China) and then was separated by 12% SDS-PAGE and transferred to PVDF nitrocellulose membrane (Invitrogen, Shanghai, China) using iblot-2 dry blotting system (Invitrogen, Shanghai, China). A protein transferred membrane blocked with 5% BSA-PBST were incubated with primary antibodies such as anti-GAPDH, anti-Col1α2 and anti-Runx2 (ABcam, Shanghai, China) overnight at 4 °C. Then the membrane was incubated with secondary goat anti-rabbit IgG-HRP (Abcam) for 1 h at 37 °C and exposed to an enhanced chemiluminescent reagent. Images were captured with a Universal Hood II Gel Doc System (Bio-Rad, Rochester, NY, USA).

### 3.10. Statistical Analysis

All the experiments were conducted three times and the data were presented as mean with standard error based on descriptive statistics. One way ANOVA (*p* < 0.05) and post hoc test were adapted using the statistical software SPSS version 16.0 (SPSS, Chicago, IL, USA).

## 4. Conclusions

In this study, two types of collagens, PSC and ASC were isolated and purified from blue shark skin, which contained three distinct chains, 2 alpha, and beta chains. The amino acid composition and the FTIR secondary structure of PSC varied with ASC due to pepsin hydrolysis. Among two collagens, PSC had high thermal stability, which might be due to high imino acid content. Most importantly, the obtained collagens promoted osteoblast cell growth and upregulated collagen synthesis in bone cells, which is a most desirable property of biomaterials for the treatment of a bone disorder. Proliferation of differentiated osteogenic cells by blue shark skin collagen may be achieved through activation of Runx2 dependent FAK/JNK signaling pathway. Overall, the blue shark skin collagens with good biochemical and osteogenic properties may be considered as a potential drug in biomedical application. Conversely, the in vivo evaluation of blue shark skin collagen and its molecular interaction with bone cells needs to be addressed by further research in order to understand the actual mechanism of the action.

## Figures and Tables

**Table 1:** The amino acid composition of ASC and PSC from blue shark skin.

Amino Acids	ASC	PSC
Glycine	392.44	387.12
Alanine	144.59	130.88
Proline	132.98	124.46
Hydroxyproline	80.45	98.28
Glutamic acid	69.23	71.50
Aspartic acid	36.73	40.51
Arginine	28.64	26.25
Leucine	24.44	13.83
Serine	21.75	26.25
Lysine	14.84	15.95
Phenylalanine	13.63	14.02
Methionine	10.17	12.03
Threonine	10.04	8.19
Histidine	5.39	6.65
Valine	7.61	8.38
Isoleucine	3.79	9.38

**Table 2:** List of primers and its sequence used in this study.

S.No	Primers Name	Primers Sequence
1	Alkaline phosphatase (ALP)	5′-TCC TGA CCA AAA ACC TCA AAG G-3′
		5′-TGC TTC ATG CAG AGC CTG C-3′
2	Osteocalcin	5′-CTC ACA GAT GCC AAG CCC-3′
		5′-CCA AGG TAG CGC CGG AGT CT-3′
3	Collagen 1 alpha 1 (Col1a1)	5′-GCG AAG GCA ACA GTC GCT-3′
		5′-CTT GGT GGT TTT GTA TTC GAT GAC-3′
4	Runx2	5′-CCA CCA CTC ACT ACC ACA CG-3′
		5′-TCA GCG TCA ACA CCA TCA TT-3′
5	Beta-actin	5′-CTG GCA CCA CAC CTT CTA CA-3′
		5′-GGT ACG ACC AGA GGC ATA CA-3′
